# Special Issue “Novel Strategies in the Development of New Therapies, Drug Substances and Drug Carriers, 3rd Edition”

**DOI:** 10.3390/ijms26199360

**Published:** 2025-09-25

**Authors:** Andrzej Kutner, Geoffrey Brown, Enikö Kallay

**Affiliations:** 1 Department of Drug Chemistry, Pharmaceutical and Biomedical Analysis, Faculty of Pharmacy, Medical University of Warsaw, 1 Banacha, 02-097 Warsaw, Poland; 2 Department of Biomedical Sciences, School of Infection, Inflammation, and Immunology, College of Medicine and Health, University of Birmingham, Birmingham B15 2TT, UK; g.brown@bham.ac.uk; 3 Department of Pathophysiology and Allergy Research, Center of Pathophysiology, Infectiology & Immunology, Medical University of Vienna, Währinger Gürtel 18-20, A-1090 Vienna, Austria; enikoe.kallay@meduniwien.ac.at

The urgent demand for effective and safe therapies—particularly for aggressive cancers [1], where delays in diagnosis and treatment can turn manageable conditions into fatal ones—continues to drive innovation in drug research. Key areas of current focus include the development of novel therapeutic molecules [2], advanced drug delivery systems [3], personalized medicine [4], and the integration of digital technologies into treatment strategies [5]. Progress in these domains requires the close integration of multiple scientific disciplines and industrial sectors, ensuring a comprehensive approach to drug discovery and development.

The first edition of the Special Issue “Novel Strategies in the Development of New Therapies, Drug Substances, and Drug Carriers” (Volume I) was published in IJMS with great success, comprising 21 papers. Volume 2.0, dedicated to the Interdisciplinary Conference on Drug Sciences, ACCORD 2022, included 25 papers and achieved similar success. Both volumes were subsequently published as MDPI printed books.

Encouraged by the continued interest of authors and readers, we reopened this topic as Volume 3.0 in *IJMS* (ISSN 1422-0067, IF 4.9, JCR Category Q1). This third edition, dedicated to the Interdisciplinary Conference on Drug Sciences, ACCORD 2024, aims to highlight research that has contributed to the conception, design, and development of new drug substances and formulations. As in the previous volumes, no thematic restrictions were imposed, enabling coverage of the full spectrum of drug development at the molecular level, consistent with the journal’s aims and scope.

The topics explored in this issue include the identification of novel molecular drug targets [2], evaluation of drug–protein interactions [6], modeling and optimization of functional activity [7], pre-formulation studies [8], pharmaceutical carrier design [9], and preclinical investigations [10]. The Special Issue assembles a diverse collection of innovative studies that span the critical stages of drug discovery, formulation, targeted delivery [9], and therapeutic monitoring [11]. Together, these contributions address distinct challenges in modern pharmacology, advancing our collective capacity to design safer and more effective treatments.

This Special Issue compiles fourteen original and review articles. They cover the entire spectrum of contemporary drug research, illustrating how complementary strategies can converge to deliver safer, more effective, and increasingly personalized therapies. The articles are organized into ten thematic groups, with each reflecting key stages of the drug development process.

1. Stability and Formulation Approaches

Witkowska et al. addressed the redox sensitivity of hydrazone-based active pharmaceutical ingredients using electrochemistry, high-resolution mass spectrometry, and Density Functional Theory to anticipate and characterize transformation products critical for manufacturability and shelf life. Pindelska et al. explored cocrystallization of febuxostat with aromatic and aliphatic amide coformers, achieving notable solubility gains despite thermal stability limitations.

2. Prodrugs and Targeted Activation

Yukimura et al. developed Ns-Dox, a glutathione (GSH)-responsive doxorubicin prodrug that is selectively activated in high-GSH cancer cells, combining potent cytotoxicity with reduced environmental toxicity.

3. Bioactive Scaffolds and Receptor-Targeted Agents

Danton et al. introduced chiral thiazoloisoindolinone derivatives as potent inhibitors of human farnesyltransferase, while Hoffmann et al. reported the stereoselective synthesis of a perhydroquinoxaline derivative exhibiting moderate κ-opioid receptor specificity. The biological activity of both compounds is supported by structural and binding studies.

4. Theranostics and Nanotechnology Platforms

Loureiro et al. presented bombesin-based theranostic modules for PET imaging and UniCAR T-cell targeting in prostate cancer, demonstrating enhanced stability and dual diagnostic–therapeutic utility. Stolarczyk et al. described diosgenin-functionalized gold nanoparticles with selective cytotoxicity toward cancer cells and provided detailed physicochemical characterization.

5. Precision Therapy for Solid Tumors

Bates et al. investigated vanadium-based Schiff base complexes with improved stability and glioblastoma-selective cytotoxicity, supporting their potential for localized, intratumoral application.

6. Multi-Target Neurotherapeutics

Wróbel et al. designed multifunctional antidepressant candidates targeting serotonin and dopamine systems, yielding two lead structures with promising in vitro activity.

7. Targeting the Cancer Microenvironment

Nakhchi et al. showed that endothelial Rap1B deletion enhanced CD8^+^ T-cell infiltration, identifying this protein as a potential immunotherapy target with reduced systemic effects.

8. Molecular Insights for Retinoid Therapies

Powała et al. elucidated ligand-binding modes of selective agonists and antagonists to retinoic acid receptor γ (RARγ), informing the rational design of retinoids with optimized specificity and pharmacokinetics.

9. Bioanalytical Tools for Therapeutic Monitoring

Kocór, Czajkowska et al. validated dried serum spots and volumetric absorptive microsampling for long-term monitoring of (val)ganciclovir in transplant patients. Kocur, Mikulska et al. review best practices for quantifying methotrexate polyglutamates to support adherence and exposure assessment.

10. Clinical Case-Based Learning

Sabbadin et al. described pseudohyperaldosteronism that was linked to excessive licorice intake, underscoring the importance of thorough dietary and medication histories in hypertensive, hypokalemic patients and highlighting the underrecognized endocrine effects of glycyrrhetinic acid.

From stability optimization and prodrug activation to nanocarrier engineering, molecular targeting, advanced analytics, and clinical application, this Special Issue offers a panoramic view of current advances in drug stability and degradation research. The Guest Editors trust that these contributions will inspire new ideas, foster cross-disciplinary collaboration, and ultimately accelerate the development of next-generation therapeutics.

## Data Availability

No new data were created.
